# Blueberry Extract and Resistance Training Prevent Left Ventricular Redox Dysregulation and Pathological Remodeling in Experimental Severe Pulmonary Arterial Hypertension

**DOI:** 10.3390/nu17071145

**Published:** 2025-03-26

**Authors:** Luciano Bernardes Leite, Leôncio Lopes Soares, Luiz Otávio Guimarães-Ervilha, Sebastião Felipe Ferreira Costa, Sara Caco dos Lúcio Generoso, Mirielly Alexia Miranda Xavier, Thainá Iasbik-Lima, Leandro Licursi de Oliveira, Ceres Mattos Della Lucia, Sara Elis Bianchi, Valquíria Linck Bassani, Flavio Gilberto Herter, Patrick Turck, Alex Sander da Rosa Araujo, Pedro Forte, Emily Correna Carlo Reis, Mariana Machado-Neves, Antônio José Natali

**Affiliations:** 1 Exercise Biology Laboratory, Department of Physical Education, Federal University of Viçosa, Viçosa 36570-900, MG, Brazil; leoncio.lopes@ufv.br (L.L.S.); sebastiao.costa@ufv.br (S.F.F.C.); sara.generoso@ufv.br (S.C.d.L.G.); anatali@ufv.br (A.J.N.); 2 Department of Sports, Instituto Politécnico de Bragança, 5300-253 Bragança, Portugal; 3 Department of General Biology, Federal University of Viçosa, Viçosa 36570-900, MG, Brazil; luiz.ervilha@ufv.br (L.O.G.-E.); mirandamirielly@gmail.com (M.A.M.X.); thaina.iasbik@ufv.br (T.I.-L.); leandro.licursi@ufv.br (L.L.d.O.); mariana.mneves@ufv.br (M.M.-N.); 4 Laboratory of Vitamin Analysis, Department of Nutrition and Health, Federal University of Viçosa, Viçosa 36570-900, MG, Brazil; cmdellalucia@ufv.br; 5 Faculty of Pharmacy, Federal University of Rio Grande do Sul, Porto Alegre 90610-000, RS, Brazil; sarabianchi@gmail.com (S.E.B.); valquiria.bassani@ufrgs.br (V.L.B.); 6 Faculty of Agronomy, Federal University of Pelotas, Pelotas 96010-610, RS, Brazil; flavioherter@gmail.com; 7 Department of Physiology, Basic Sciences Institute of Health, Federal University of Rio Grande do Sul, Porto Alegre 90035-003, RS, Brazil; p.turck@gmail.com (P.T.); alex.rosa@ufrgs.br (A.S.d.R.A.); 8 Department of Sports, Higher Institute of Educational Sciences of the Douro, 4560-708 Penafiel, Portugal; 9 CI-ISCE, ISCE Douro, 4560-547 Penafiel, Portugal; 10 Research Center for Active Living and Wellbeing (LiveWell), Instituto Politécnico de Bragança, 5300-253 Bragança, Portugal; 11 Department of Veterinary Medicine, Federal University of Viçosa, Viçosa 36570-900, MG, Brazil; emily.carlo@ufv.br

**Keywords:** pulmonary artery resistance, exercise training, left ventricle, redox state, ejection fraction, adverse remodeling, blueberry extract

## Abstract

Objective: To investigate whether the regular administration of blueberry extract and low-intensity resistance exercise training (RT), either alone or in combination, during the development of monocrotaline (MCT)-induced severe pulmonary arterial hypertension (PAH) in rats protect the left ventricle (LV) from redox dysregulation and pathological remodeling. Methods: Groups of seven male Wistar rats were formed for the experiment: sedentary control; sedentary hypertensive; sedentary hypertensive blueberry; exercise hypertensive; and exercise hypertensive blueberry. PAH was experimentally induced through a single intraperitoneal administration of MCT at a dose of 60 mg/kg. One day after injection, the blueberry groups started receiving a daily dose of blueberry extract (100 mg/kg) by gavage, while the exercise groups initiated a three-week program of RT (ladder climbing; 15 climbs carrying 60% of maximum load; one session/day; 5 times/week). Echocardiographic evaluations were conducted 23 days after injection, and the rats were euthanized the next day to harvest LV tissue. Results: Separately, blueberry extract and RT mitigated augments in pulmonary artery resistance, LV tissue redox dysregulation (i.e., increased PC levels) and detrimental remodeling (i.e., reduced inflammation), and reductions in ejection fraction (EF) and fractional shortening (FS) caused by PAH. The combination of treatments prevented reductions in EF and FS, along with the development of a D-shaped LV. Conclusions: blueberry extract and moderate-intensity resistance training administered during the development of MCT-induced severe PAH in rats prevented LV redox dysregulation and pathological remodeling, thereby preserving its function.

## 1. Introduction

Pulmonary arterial hypertension (PAH) is primarily a right heart disease [1], and disruptions in redox homeostasis—characterized by increased reactive species production alongside weakened antioxidant defenses—are considered key contributors to its development [2]. However, in patients with severe PAH, mortality has been independently associated with left ventricular (LV) global longitudinal strain [3]. In this context, LV dysfunction has been observed in these patients, marked by impaired LV filling and a reduction in end-diastolic volume [4,5,6,7]. These alterations are thought to stem from changes in LV geometry, such as the development of a D-shaped configuration [7,8], driven by right ventricular (RV) hypertrophy, dilation, and interventricular septal flattening [9]. These structural modifications affect LV segmental function and torsion, ultimately leading to a reduced systolic volume [10,11,12,13,14].

The rat model of monocrotaline (MCT)-induced severe PAH (i.e., 60 mg/kg) has contributed over the years to the understanding of the disease’s development and potential therapy proposals. The involvement of oxidative stress in the development of right ventricular (RV) dysfunction is evident in this model, with MCT inducing the elevated production of reactive oxygen species (ROS) and triggering proapoptotic pathways [7,15,16]. These mechanisms disrupt calcium cycling by affecting its regulatory proteins, ultimately leading to contractile impairment [7,17,18]. Recently, our research group demonstrated that LV dysfunction also occurs in rats with severe PAH induced by MCT, as redox homeostasis was disrupted [i.e., reduced catalase (CAT) and superoxide dismutase (SOD); and increased malondialdehyde (MDA)], and adverse functional and structural remodeling were observed [7]. Therefore, under the condition of oxidative stress, potential therapies that stimulate the increase in cellular antioxidant capacity are of interest [19].

In this background, alternative therapeutic strategies like the use of natural antioxidants and physical exercise have been investigated. For instance, our group showed that blueberry (*Vaccinium myrtillus*) extract, which is rich in polyphenols, especially anthocyanins, restored the redox state and contributed to attenuating pulmonary pressure on the lungs of rats with MCT-induced severe PAH [20]. Similarly, blueberry extract lessened the RV detrimental remodeling, lipid peroxidation, and nicotinamide adenine dinucleotide phosphate (NADPH) oxidase activity and increased CAT activity in this model [21]. Moreover, blueberry (*Vaccinium* spp.) has been proven to reduce LV fibrosis and inflammation in a cardiac injury model due to improvements in redox balance [22]. Regarding physical exercise, we have demonstrated that aerobic and combined (i.e., resistance plus aerobic) types are beneficial to mitigate oxidative stress, and adverse remodeling contractile parameters in both RV and LV in rats with MCT-induced severe PAH, which results in the maintenance of cardiac function and physical exercise tolerance [7,23].

Therefore, considering that approaches involving natural antioxidant fruits and resistance exercise training (RT) have been sparsely explored in severe PAH, especially aiming at the LV, we thought to investigate whether the regular administration of blueberry extract and moderate-intensity RT, either alone or in combination, during the development of MCT-induced severe PAH in rats, protect the LV from redox dysregulation and pathological remodeling.

## 2. Materials and Methods

### 2.1. Animals

**Figure 1:**
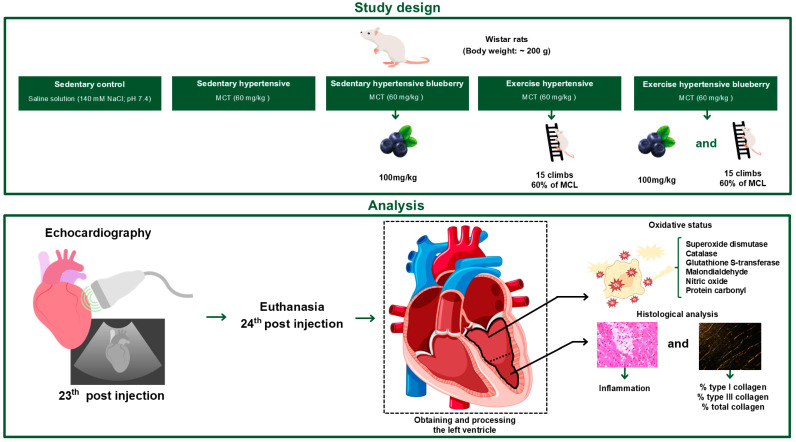
Flowchart of the study design with its respective analyses.

Male Wistar rats (body weight: ~200 g) were housed in transparent polycarbonate cages, four animals per cage. The animals were divided into five experimental groups, with seven animals per group: sedentary control (SC), sedentary hypertensive (SH), sedentary hypertensive blueberry (SHB), exercise hypertensive (EH), and exercise hypertensive blueberry (EHB). All animals had free access to tap water and commercial chow, and environmental conditions were kept constant [temperature of 23 ± 1 °C, light/dark regime of 12/12 h, and relative humidity of approximately 60%]. This study was conducted in compliance with ethical standards and was approved on 8 July 2022 by the Ethics Committee on the Use of Animals at the Federal University of Viçosa under protocol number 11/2022. Figure 1 presents a flowchart of the study design with its respective analyses.

### 2.2. PAH Induction

The animals in the SH, SHB, EH, and EHB groups were administered a single intraperitoneal injection of MCT (60 mg/kg body weight) (Sigma-Aldrich, St. Louis, MO, USA) dissolved in a saline solution (140 mM NaCl; pH 7.4) [24]. The animals in the SC group were administered an equivalent volume of saline solution (140 mM NaCl; pH 7.4).

### 2.3. Preparation and Characterization of Blueberry Extract

Freshly ripened blueberries (cultivar Bluegem, group Rabbiteye) were harvested manually by gently handpicking each berry from its stem and storing them in a sterile plastic bag stored in dry ice. The extraction process combined 200 g of fresh blueberries with 500 mL of a 50% (*v*/*v*) ethanol extraction solvent. The mixture underwent initial extraction in a cutting mill for 10 min, followed by sonication for 30 min in an ultrasound bath containing ice to avoid compound degradation. The resulting mixture was then vacuum filtered to separate the extract. The extract was further concentrated to 100 mL using a rotary evaporator.

### 2.4. Composition and Administration of Blueberry Extract

The extract contained a total phenolic compound concentration of 7795.48 mg gallic acid equivalent (GAE) per liter. After processing, the concentration of phenolics was 38.89 mg GAE/g of extract. As for the identification and concentrations of anthocyanidins, the following were observed: cyanidin (219.21 µg/mL) and malvidin (128.68 µg/mL). Other phenolic compounds (rutin, gallic acid, catechin, and epicatechin) were not detected (Table 1). Considering the composition of the extract, the rats in the SHB and EHB groups received a daily dose of blueberry extract (100 mg/kg of animal body weight) administered by gavage at 7:00 a.m. during the experimental protocol. This amount was calculated based on the average amount of total polyphenols consumed by populations in North and South America [25] and has been shown to have a beneficial effect on the treatment of experimental severe PAH [20,21].

### 2.5. Blueberry Extract Analysis

This study employed high-performance liquid chromatography (HPLC) featuring a SIL-20A autosampler, an HPLC-20AT pump, and an SPD-20AV photodiode array detector (Shimadzu HPLC-20A, Kyoto, Japan). Data acquisition was performed using Shimadzu HPLC Solution GPC software version 2.03 (Shimadzu, Kyoto, Japan). The stationary phase comprised a CLC-ODS column (4.6 mm × 25 cm i.d.; 5 μm) (Shimadzu, Kyoto, Japan) combined with a C18 pre-column (20 mm × 3.9 mm i.d.; 10 μm) (Waters, Mil-ford, MA, USA). The mobile phase consisted of solvent A (ultrapure water with 0.1% trifluoroacetic acid) and solvent B (acetonitrile), applied under a gradient elution: 8% B for 5 min, increasing from 8% to 25% B over 25 min, maintaining 25% B for 5 min, and then returning to 8% B over 10 min. The flow rate was set at 0.8 mL/min, with a detection wavelength of 515 nm and an injection volume of 20 μL. Before injection, all samples were filtered through a Millipore polytetrafluoroethylene (PTFE) membrane with a 0.45 μm pore size.

### 2.6. Resistance Exercise Training

All rats underwent a two-week familiarization period with the RT model [26], during which they climbed a vertical ladder (height: 1.1 m; width: 0.18 m; step spacing: 2 cm; inclination: 80°) carrying a proportional load attached to their tail. Following this adaptation period, a maximum load test was performed. Rats began the test carrying 75% of their body weight, with 30 g added after each subsequent climb until exhaustion, with a 120 s rest interval between climbs. After completing the maximum load test and PAH induction, the EH and EHB groups participated in a four-week RT program. This consisted of 15 climbs per session, carrying 60% of their maximum load, with 60 s rest intervals between climbs, performed once daily, five days a week (Monday to Friday). Previous research has highlighted the advantages of this protocol in the context of MCT-induced PAH [7,27,28,29]. The training load was adjusted based on performance in a new maximum load test conducted 14 days after PAH induction. It is noteworthy that no signs of right ventricular failure (i.e., cyanosis, edema) were observed either during or after the exhaustive physical effort.

### 2.7. Echocardiography

Echocardiographic evaluations were performed on all animals 23 days after the MCT injection. The animals were anesthetized with 1.5% isoflurane in 100% oxygen, delivered at a constant flow rate of 1 L/min (Isoflurane, Bio-Chimico, Rio de Janeiro, Brazil). Imaging was conducted with the animals positioned in lateral decubitus. Two-dimensional echocardiographic studies were carried out at a high frame rate of 120 fps in M mode, using the MyLabTM30 ultrasound system (Esaote, Genoa, Italy) with 11 MHz transducers. Transthoracic echocardiography and M-mode scans were obtained at a scanning speed of 200 mm/s, synchronized to the heart rate.

Image acquisition adhered to the guidelines of the American Society of Echocardiography and was stored for further analysis [30]. LV function was evaluated using ejection fraction (EF) and fractional shortening (FS). Pulmonary arterial hypertension (PAH) was confirmed by calculating the ratio of acceleration time (AT) to ejection time (ET). A quantitative analysis was conducted to identify the presence or absence of a flattened interventricular septum (i.e., D-shaped left ventricle) in each animal based on echocardiographic images, as used in previous studies [7,28,29].

### 2.8. Sample Collection

All animals (SC, SH, SHB, EH, and EHB groups) were euthanized by decapitation on the 24th day following the MCT injection. Such a time point was chosen based on prior studies [7,28,29], which corresponds to the average survival period for animals with PAH induced by a 60 mg/kg dose of MCT. After euthanasia, the hearts of the animals were removed by an experienced researcher (L.L.S.), carefully dissected, and the absolute weight was obtained. Afterward, the LV was dissected and divided in half, with one portion being standardized for histological analysis and the other for enzymatic analysis (Supplementary Material). The histological procedures and enzymatic analyses are described in detail below. Also, at the time after euthanasia, the left tibia of the animals was removed and dissected, and measurements were performed to determine the heart weight/tibia length ratio.

### 2.9. Histological Processing and Histopathological Analysis

Histological analyses of the LV were performed following standardized protocols [31]. LV fragments *(n =* 7 animals/group*)*, obtained immediately after dissection, were fixed in Karnovsky’s solution (4% paraformaldehyde and 4% glutaraldehyde in 0.1 M phosphate buffer, pH 7.4) for 24 h. The fragments were then dehydrated in increasing concentrations of ethanol (70%, 80%, 90%, and 100%), cleared in xylene, and embedded in paraffin. Ten histological sections, per animal, with a thickness of 5 µm were obtained in semi-series using a rotary microtome, respecting intervals of 100 µm between consecutive sections to avoid repetition of areas from adjacent regions [32,33]. Histological sections of cardiac tissue were stained with hematoxylin and eosin (HE) for histopathological and stereological analysis and Sirius red staining (Sirius red F3B, Mobay Chemical Co., Union, NJ, USA) to quantify collagen fibers [32]. The slides were analyzed for the histopathological structure of the LV using an optical microscope (Olympus AX-70, Tokyo, Japan) at ×200 magnification. The sections were analyzed entirely by a blinded researcher (L.O.G-E), and the semi-qualitative presence of inflammation foci was analyzed according to Navarro-Hortal et al. [34]. Specifically, inflammation was assessed using a six-level scale: 0 indicated the absence of lesions, I corresponded to fewer than three foci, II to more than three foci, III to mild generalized inflammation, IV to moderate inflammation, and V to severe inflammation. Representative images of the LV were captured using a coupled digital camera (Olympus Q Color-3, Tokyo, Japan).

### 2.10. Quantification of Collagen Fibers

Sirius red sections were analyzed in ten histological fields per animal (*n* = 7 animals/group) using Image-Pro Plus 4.5^®^ software (Media Cybernetics, Silver Spring, MD, USA) to quantify collagen fibers. A polarized filter was attached to the microscope to identify collagen fibers in Sirius red-stained sections. Pixels identified as type I (orange-red) and type III (yellowish-greenish) collagen were quantified based on the software’s color histogram [33]. Results were expressed as a percentage of the total image area (1.92 × 10^6^ µm^2^), with the sum of the percentages of both collagen types representing the total amount of fibers.

### 2.11. Evaluation of Antioxidant Enzyme Activity and Oxidative Stress Indicators

LV samples (*n* = 6/group) stored at −80 °C (~100 mg) were homogenized in 1 mL of phosphate buffer (pH 7.4) and centrifuged at 10,000× *g* for 10 min at 4 °C. The supernatants were used to evaluate the enzymatic activity of SOD, CAT, and glutathione S-transferase, and to quantify the byproducts of nitrosative and oxidative stress, specifically nitric oxide and MDA, along with total protein quantification. The resulting pellets were used to determine carbonylated proteins. All analyses were performed using an ELISA microplate reader (Multiskan SkyHigh, Thermo Scientific, Waltham, MA, USA).

#### 2.11.1. Superoxide Dismutase

SOD activity was assessed based on its ability to catalyze the conversion of superoxide (O^2−^) into hydrogen peroxide (H_2_O_2_), following the pyrogallol auto-oxidation method [35]. The reaction mixture contained 10 μL of the sample and 170 μL of sodium phosphate buffer (pH 7.8). The reaction was initiated by adding 20 μL of pyrogallol (10 mM) and incubated at 37 °C for 30 min. Absorbance was measured at 320 nm. SOD activity was expressed in units (U)/mg of protein, with one unit of SOD defined as the amount needed to inhibit the pyrogallol auto-oxidation rate by 50%.

#### 2.11.2. Catalase

CAT activity was assessed by measuring the rate of hydrogen peroxide (H_2_O_2_) decomposition, following the method described by Aebi [36]. Briefly, 100 μL of H_2_O_2_ (20 mM) was added to 5 μL of the sample. After 3 min, 150 μL of ammonium molybdate (32.4 mM) was added to terminate the reaction. Sample blanks were prepared by replacing H_2_O_2_ with sodium phosphate buffer (50 mM, pH 7.4). The values obtained from the test samples were adjusted by subtracting the blank values. A standard curve was generated using serial dilutions of H_2_O_2_ to determine CAT activity. Absorbance was measured at 374 nm using a spectrophotometer. CAT activity was expressed in units (U)/mg of protein, with one unit defined as the amount of enzyme required to decompose 1 mmol of H_2_O_2_ per minute.

#### 2.11.3. Glutathione S-Transferase

Glutathione S-transferase (GST) activity was evaluated using the method described by Habig et al. [37], measuring the formation of glutathione conjugated to 2,4-dinitrochlorobenzene (CDNB). The reaction mixture consisted of 0.1 M CDNB, 0.1 M reduced glutathione (GSH), and sodium phosphate buffer (0.1 M; pH 7.2), along with 10 μL of the sample. After the addition of CDNB, the reaction progress was monitored by measuring the absorbance change at 340 nm over 60 s, with readings taken at two intervals (30 and 90 s). The molar extinction coefficient for CDNB was ε340 = 9.6 mmol L^−1^ cm ^−1^. GST activity was expressed in units (U)/mg of protein, where one unit of GST corresponds to the amount of enzyme that catalyzes the formation of 1 μmol of product per minute per milliliter.

#### 2.11.4. Nitric Oxide

Nitric oxide (NO) production was quantified using the Griess reaction, following the method described by Tsikas [38]. Samples (50 μL) were incubated with an equal volume of Griess reagent (50 μL; 1% sulfanilamide, 0.1% N-(1-naphthyl)ethylenediamine, and 2.5% H_3_PO_4_) at room temperature for 10 min. NO concentration was determined using a sodium nitrite standard curve (0–125 μM) and expressed in μmol L^−1^. Absorbance was measured at 570 nm.

#### 2.11.5. Malondialdehyde

Lipid peroxidation was assessed by quantifying total malondialdehyde (MDA) following the method of Buege and Aust [39]. Briefly, 200 μL of the sample supernatant was mixed with 400 μL of TBARS solution (15% trichloroacetic acid, 0.375% thiobarbituric acid, and 0.25 M HCl). The reaction mixture was incubated in a water bath at 90 °C for 40 min. After cooling on ice, 600 μL of butyl alcohol was added, and the solution was shaken and centrifuged at 3500 rpm for 5 min. A standard curve using known concentrations of 1,1,3,3-tetramethoxypropane (TMPO) was applied to calculate MDA concentration. The formation of thiobarbituric acid-reactive substances was monitored by absorbance at 535 nm, and the results were expressed as nmol/mg of protein.

#### 2.11.6. Carbonylated Proteins

Protein oxidation was evaluated using the method of Levine et al. [40] by quantifying the carbonyl groups present in the protein structure. The concentration of carbonylated proteins (CPs) was determined by the reaction of carbonyl groups with 2,4-dinitrophenylhydrazine (DNPH). The pellets obtained from the sample centrifugation were resuspended in 0.5 mL of DNPH solution (10 mM) diluted in 2 M HCl, vortexed, and incubated at room temperature in the dark for 30 min with periodic shaking. To stop the reaction, 0.5 mL of ice-cold 10% trichloroacetic acid (TCA) was added, and the mixture was centrifuged at 5000× *g* for 10 min at 4 °C. The supernatant was discarded, and the precipitate was washed three times with 1 mL of a 1:1 (*v*/*v*) mixture of ethyl acetate and ethanol, followed by centrifugation. Next, 1 mL of 6% sodium dodecyl sulfate (SDS) was added, and the mixture was centrifuged. The absorbance of the supernatant was measured at 370 nm. The results were expressed as nmol/mL, based on a molar extinction coefficient of ϵ370 = 22 mmol/L/cm.

#### 2.11.7. Total Protein

Total protein quantification was performed using the Bradford method [41], with bovine serum albumin as the standard. Protein content was used to normalize the results for SOD, CAT, GST, and MDA.

### 2.12. Statistical Analysis

The Shapiro–Wilk test was used to assess data distribution. All data were parametric and analyzed using one-way analysis of variance (ANOVA) to identify differences between groups, followed by Tukey’s post hoc test for multiple comparisons when applicable. Fisher’s exact test was used to evaluate the proportion of animals exhibiting flattening of the interventricular septum. Pairwise comparisons between groups were performed as appropriate. Quantitative results are expressed as mean ± SEM, while qualitative data are presented as percentages. A significance threshold of 5% (*p* ≤ 0.05) was adopted for all statistical tests. The specific tests and the number of animals analyzed for each parameter are detailed in the corresponding figures. All statistical analyses were performed using GraphPad Prism Software version 10.4.0.

## 3. Results

### 3.1. Validation of Pulmonary Hypertension

A qualitative analysis of the echocardiographic images (Supplementary Figure S1A) shows that all hypertensive groups (SH, SHB, EH, EHB) exhibited impairments in pulmonary artery flow dynamics, indicating hemodynamic changes compared with the SC group. However, in the treated groups (SHB, EH, and EHB), the pulmonary artery flow was visibly less altered compared with SH, suggesting a protective effect of the interventions. These observations were confirmed by the quantitative analysis of the TA/TE ratio, a hemodynamic index of pulmonary arterial flow. Animals in the SH group had lower TA/TE values (0.22 ± 0.01) than those in the SC group (0.61 ± 0.02), reinforcing the pulmonary vascular impairment induced by PAH. However, the SHB (0.39 ± 0.01), EH (0.45 ± 0.02), and EHB (0.44 ± 0.02) groups showed higher TA/TE values than SH, confirming the beneficial effects of blueberry and exercise.

### 3.2. LV Echocardiographic Data

**Figure 2:**
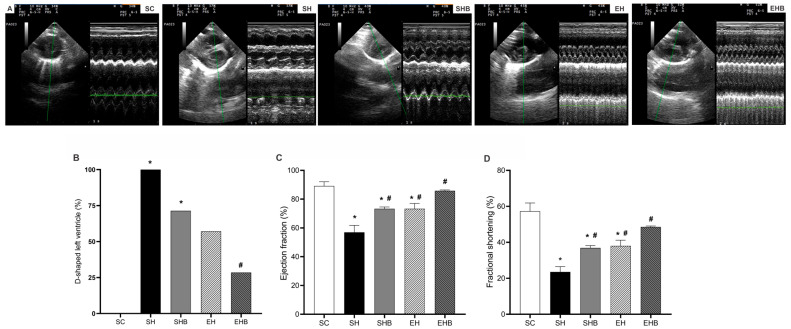
Effects of blueberry extract and resistance training on left ventricular function. (**A**) Representative echocardiograph images. (**B**) D-shaped left ventricle. (**C**) Ejection fraction. (**D**) Fractional shortening. The data are expressed as means ± SEM of seven rats per group. SC—sedentary control; SH—sedentary hypertensive; SHB—sedentary hypertensive blueberry; EH—hypertensive exercise; EHB—exercise hypertensive blueberry. * *p* < 0.05 vs. SC; # *p* < 0.05 vs. SH. Panel (**A**) (Fisher’s exact test). Panel (**B**,**C**) (one-way ANOVA followed by Tukey’s post hoc test).

Rats in the SH and SHB groups showed greater interventricular septal flattening (D-shaped LV) compared with those in the SC group (Figure 2A). Although blueberry ex-tract (SHB group) alone did not affect this alteration, the combination of treatments (EHB group) prevented flattening. Moreover, animals with PAH and not treated (SH group) presented reduced EF (Figure 2B) and FS (Figure 2C) compared with the controls (SC group). Both treatments (SHB and EH groups) alleviated these reductions, and the combination of treatments (EHB group) maintained EF and FS values similar to those of the control group.

### 3.3. Morphology and Adverse Remodeling of the LV Tissue

**Figure 3:**
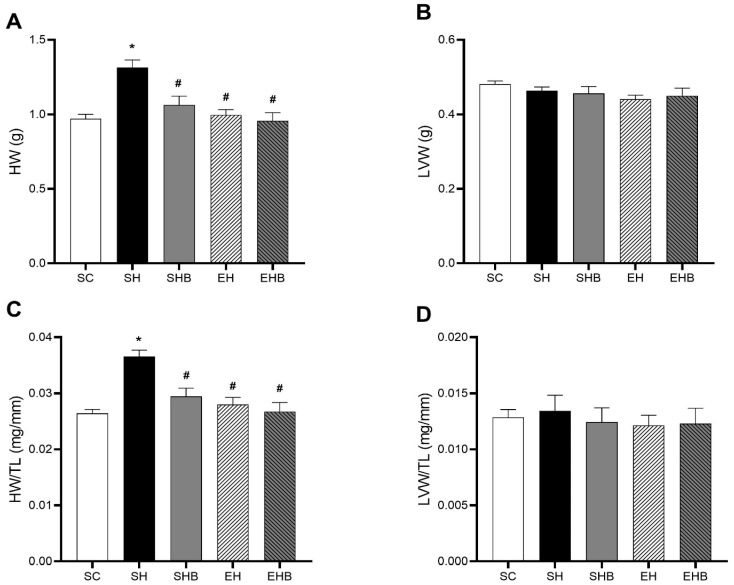
Effects of blueberry extract and resistance training on cardiac morphometric parameters. (**A**) Heart weight (HW). (**B**) Left ventricular weight (LVW). (**C**) Heart weight/tibial length (HW/TL). (**D**) Left ventricular weight/tibial length (LVW/TL). The data are expressed as means ± SEM of seven rats per group. SC—sedentary control; SH—sedentary hypertensive; SHB—sedentary hypertensive blueberry; EH—hypertensive exercise; EHB—exercise hypertensive blueberry. * *p* < 0.05 vs. SC; # *p* < 0.05 vs. SH. One-way ANOVA followed by Tukey’s post hoc test.

Animals injected with MCT and not treated (SH group) showed higher heart weight (HW; Figure 3A) and heart weight/tibia length ratio (HW/TL; Figure 3C) than the controls (SC group). Nevertheless, both treatments, either isolated (SHB and EH groups) or in combination (EHB group), hindered these increases. There were no differences between groups in LV weight (Figure 3B) and LV weight/tibia length ratio (Figure 3D).

**Figure 4:**
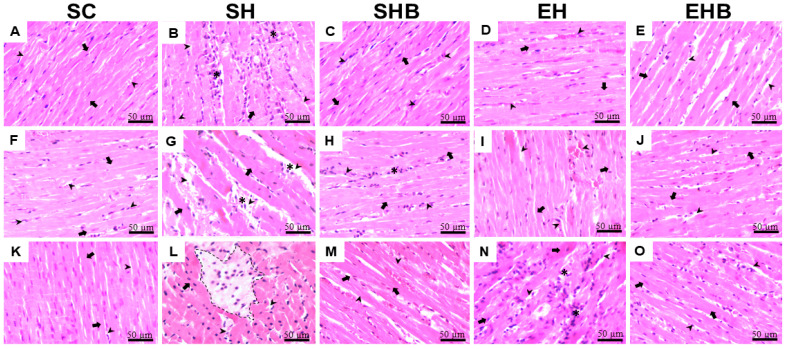
(**A**–**O**) Representative photomicrographs of left ventricle tissue stained with hematoxylin and eosin (scale bar: 50 μm). SC—sedentary control; SH—sedentary hypertensive; SHB—sedentary hypertensive treated with blueberry extract; EH—hypertensive treated with resistance training; EHB—hypertensive treated with combined blueberry extract and resistance training. Black arrows: cardiomyocytes; arrowheads: extracellular matrix; asterisks: inflammatory foci; dotted regions: areas of cell death. Data are expressed as means ± SEM of seven rats per group.

The qualitative analysis revealed that healthy animals from the SC group exhibited preserved cardiac tissue architecture (Figure 4A,F,K and Figure S1B), with aligned cardiomyocytes, well-defined central nuclei, and homogeneous cytoplasm, without signs of inflammation, cell death, or abnormal extracellular matrix accumulation. In contrast, animals injected with MCT and not treated (SH group) showed disorganized cardiac muscle fibers, reduced cardiomyocyte area, and increased extracellular matrix (Figure 4B,G,L and Figure S1B), along with frequent cellular infiltrates (Table 2) and areas of cell death, indicating severe inflammation and structural damage.

Animals receiving isolated treatment with blueberry extract (SHB group) presented fewer inflammatory foci and lower cellular infiltrate density (Figure 4C,H,M and Supplementary Figure S1B; Table 2), with partial preservation of muscle fibers and reduced extracellular matrix compared with the untreated animals (SH group) (Figure 4C,H,M). Similarly, animals undergoing the isolated treatment with resistance training (EH group) showed improved muscle fiber organization, reduced inflammation, and partial preservation of cardiomyocyte and extracellular matrix (Figure 4D,I,N and Figure S1B; Table 2). Finally, animals submitted to the combined treatment (EHB group) demonstrated more evident preservation of LV morphology, with fewer inflammatory foci and maintained muscle fiber architecture (Figure 4E,J,O and Figure S1B). None of the treated groups (SHB, EH, and EHB) exhibited areas of cell death (Figure 4 and Figure S1B), highlighting the protective effect of the interventions.

**Figure 5:**
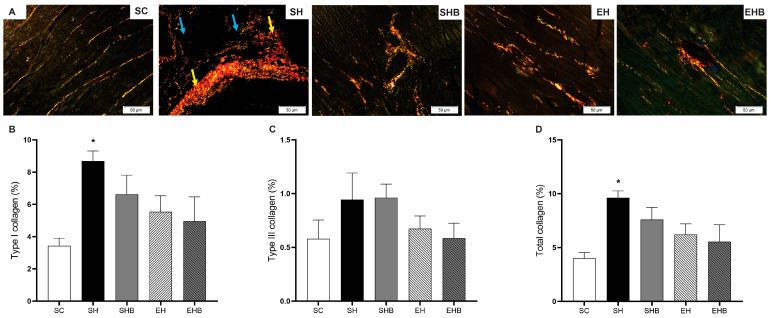
Effects of blueberry extract and resistance training on left ventricular collagen deposition. (**A**) Representative photomicrographs of LV tissue stained with Sirius red. (**B**) Percentage of type I collagen. (**C**) Percentage of type III collagen. (**D**) Percentage of total collagen. The data are expressed as means ± SEM of seven rats per group. SC—sedentary control; SH—sedentary hypertensive; SHB—sedentary hypertensive blueberry; EH—hypertensive exercise; EHB—exercise hypertensive blueberry. Yellow arrows: Type I collagen; blue arrows: Type III collagen. One-way ANOVA followed by Tukey’s post hoc test. * *p* < 0.05 vs. SC.

Figure 5A presents representative photomicrographs of LV tissue, illustrating notable structural differences among the experimental groups. The SH group exhibited pronounced collagen deposition, as indicated by the presence of type I collagen (yellow arrows) and type III collagen (blue arrows), in contrast with the other groups, which displayed a more preserved myocardial architecture. Figure 5B,D quantitatively confirm these observations. Hypertensive untreated rats (SH group) exhibited a significantly higher percentage of type I collagen (Figure 5B) and total collagen (Figure 5D) compared with the control animals (SC group) (*p* < 0.05), suggesting increased fibrosis. However, no significant differences were observed in type III collagen levels among the groups (Figure 5C). Nonetheless, the protective effect of treatments, either isolated or in combination, did not reach statistical significance.

### 3.4. Oxidative Stress Markers

**Figure 6:**
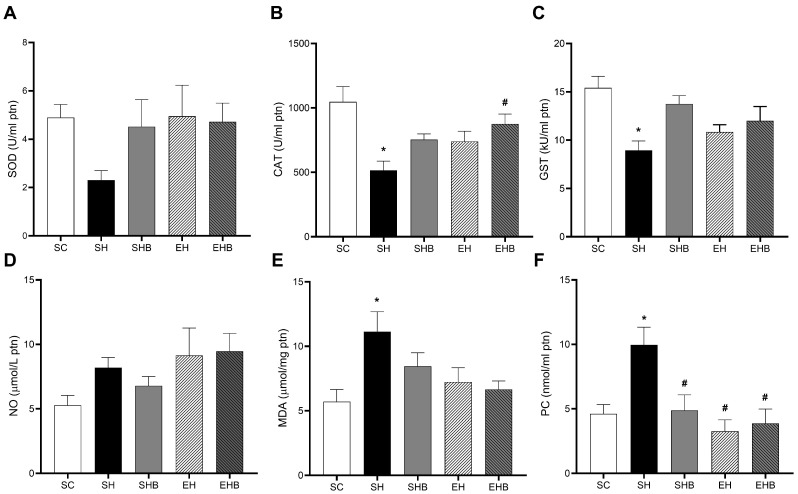
Effects of blueberry extract and resistance training on left ventricular oxidative stress biomarkers. (**A**) CAT (catalase), (**B**) SOD (superoxide dismutase), (**C**) GST (glutathione S-transferase), (**D**) MDA (malondialdehyde), (**E**) NO (nitric oxide), (**F**) PC (protein carbonyl). The data are expressed as means ± SEM for five or seven rats per group. SC—sedentary control; SH—sedentary hypertensive; SHB—sedentary hypertensive blueberry; EH—hypertensive exercise; EHB—exercise hypertensive blueberry. One-way ANOVA followed by Tukey’s post hoc test. * *p* ˂ 0.05 vs. SC; # *p* ˂ 0.05 vs. SH.

The reduction in SOD activity caused by MCT injection did not reach statistical significance (Figure 6A), neither did the effects of the treatments. The rats in the SH group showed lower CAT activity than those in SC (Figure 6B). While isolated treatments (SHB and EH groups) did not affect significantly such dysregulation, the combined treatments avoided such a reduction (SH < EHB), which suggests a synergistic action of treatments. The activity of GST was also decreased by MCT injection (SH < SC); however, no effects of treatments were observed (Figure 6C). The concentrations of NO were not changed by MCT, and no significant effect of treatments were observed (Figure 6D). Regarding MDA (Figure 6E), MCT increased its concentrations (SH > SC); however, no significant protective effects of treatments were found. The rats in the SH group showed higher PC levels compared with those in the SC group (Figure 6F). Nevertheless, both treatments, either isolated (SHB and EH groups) or combined (EHB group), protected the hypertensive rats from such increases to a similar extent.

## 4. Discussion

This study investigated whether the regular administration of blueberry extract and moderate-intensity RT, either alone or in combination, during the development of MCT-induced severe PAH in rats, protect the LV from redox dysregulation and pathological remodeling. We found that both treatments effectively prevented increases in pulmonary artery resistance, LV deformation, redox dysregulation, and adverse remodeling, thereby preserving LV function.

The MCT injection used in the present study increased the pulmonary artery resistance, which directly imposed an overload to the RV and flattened the interventricular septum. Consequently, it imposed LV’s morphological, structural, and functional damages. To illustrate, both EF and FS were reduced in rats from the SH group. Such effects have been revealed in a previous study on this model [7]. It is reasonable that such effects are caused by morphological, structural, and redox balance changes. In fact, our hypertensive rats who were not treated had D-shaped LV and cardiac hypertrophy, which can negatively influence LV segmental function and torsion and thus decrease the systolic volume [42,43]. In addition, these sedentary hypertensive rats exhibited augmented LV tissue ECM, type I, and total collagen, along with evidence of inflammation (i.e., increased cellular infiltrate and cell death). Moreover, these rats’ LV tissue presented lower activities of CAT and GST, while the concentrations of MDA and PC were higher compared with the control animals. Such LV tissue redox dysregulation was reported previously by our group [7] and is known to be induced by MCT [44,45] as it increases the cardiopulmonary production of ROS and activates the proapoptotic pathways [6,18]. Oxidative stress induces fibroblast activation, thus accumulating collagen [46], and damages DNA, proteins, and lipids of cardiac cells causing cell death [47] and augmenting, therefore, fibrosis. MCT also leads to LV overexpression of endothelin-1, tenascin-C, and beta-myosin heavy chain and angiotensin-converting enzyme upregulation [44]. Together, these changes lead to impairments in the LV filling and end-diastolic volume [12,13,14] as they diminish the LV tissue’s elasticity. Furthermore, increased ROS can directly affect the calcium cycling regulatory proteins [48,49] and impair LV myocyte contractile function, as recently reported by our group [7,28].

More importantly, the employed treatments prevented increases in pulmonary artery resistance and interventricular septum flattening which protected the LV of hypertensive rats from the harmful changes caused by MCT. For instance, the administration of either blueberry extract or RT alone mitigated EF and FS reductions. Notably, the combination of treatments induced even greater protection and maintained these LV functional parameters to control levels. It is conceivable that such protective effects are based on morphological, structural, and redox changes promoted by the treatments. To illustrate, blueberry extract and RT, either isolated or combined, avoided cardiac hypertrophy promoted by MCT. The singular protective effects of blueberry and other antioxidants (i.e., quercetin, L-arginine, naringenin, ellagic acid, and beet juice) on cardiac hypertrophy in this model of severe PAH have been demonstrated [50,51,52,53,54], whereas the protective effect of combined exercise (i.e., RT plus aerobic exercise) was recently reported by our group [7]. We also demonstrated the beneficial effects of RT alone on LV morphology in rats with stable PAH [28]. Nonetheless, the present study reveals for the first time the beneficial effect of combining blueberry extract and RT against pathological LV remodeling in severe MCT-induced PAH.

At the LV tissue level, the structural damages induced by MCT were counteracted by the treatments. For instance, the qualitative analysis showed that the treatments, whether applied individually or in combination, similarly prevented inflammation and extracellular matrix accumulation, and promoted better tissue organization with preservation of cardiomyocytes in the LV. Regardless of no treatment’s significant effect against the augmented amount of LV collagen, the observed protection against the increase in extracellular matrix and inflammation may be due to the maintenance of redox balance in response to the treatments. Indeed, the combined treatments avoided the reduction in CAT activity, showing a synergistic action of blueberry extract and RT, since no single effects were found. Furthermore, the increase in PC levels was halted by both treatments, whether administered individually or in combination, with comparable efficacy. Previous studies on MCT-induced severe PAH have shown that single treatments with blueberry extract [21] and combined exercise training [7] can reduce oxidative stress and prevent redox dysregulation in RV and LV tissues, respectively.

The beneficial effects of blueberry on RV tissue’s redox status in the studied model seem to be related to the attenuation of increases in pro-oxidant enzymes (i.e., NADPH oxidase), though no change in the levels of O2·^−^ was found [21]. Such a study also showed that blueberry extract elevated CAT activity, though it neither restored SOD nor changed GPx activity, which resulted in improved pulmonary artery resistance (e.g., TA/TE) and RV functional parameters (e.g., CO). Concerning LV, the blueberry anthocyanin-enriched extract was shown to be efficient in reducing fibrosis, inflammation, and LV dysfunctions in a model of cyclophosphamide-induced cardiac injury. These effects were associated with protection against redox dysregulation since they increased the levels of SOD and GSH and reduced those of MDA [22].

Regarding physical exercise, the beneficial effects of aerobic exercise training on maintaining the systemic and heart redox regulation in PAH by improving the antioxidant defense are well established [4,55]. Nevertheless, RT approaches involving its antioxidant effects have rarely been explored, especially in PAH models and aimed at the LV tissue. Recently, our group examined its effects on the LV of rats with MCT-induced stable PAH [45] and found that RT mitigated the MCT damages to the LV contractile function by reducing LV collagen and fibrosis. We also demonstrated that combined exercise (RT + aerobic exercise) provided protection for the LV against structural and functional damage induced by MCT in a model of severe PAH by maintaining the activities of CAT and SOD and preventing the increase in MDA levels [7], thus avoiding redox dysregulation.

Our results show that blueberry extract and RT mitigated MCT-induced redox dysregulation, reducing pulmonary artery resistance and thus protecting against detrimental LV structural and functional remodeling during severe PAH development. While the MCT-induced PAH model mirrors key aspects of human PAH, physiological and metabolic differences between rats and humans must be considered. Nonetheless, the observed protective effects suggest promising therapeutic potential for further clinical investigations.

Lastly, the findings of our study should be interpreted with some caution, as there are a few limitations. First, we cannot completely rule out the direct effects of MCT on the LV, which may have influenced the observed improvements in LV function. Second, the chemical composition of blueberry extracts can vary depending on factors like growing, harvesting, and processing, which may affect the consistency of results when using this type of intervention. Third, protease inhibitors were not used during sample processing, which may have led to partial protein degradation and, consequently, influenced the accuracy of enzymatic activity values. Lastly, although hemodynamic impairment was confirmed by echocardiography on day 23, we neither performed serial assessments to monitor the progression of pulmonary hypertension nor were we able to calculate cardiac efficiency due to limited access to advanced imaging techniques. These aspects should be considered when interpreting the functional findings. Future studies are encouraged to analyze the effects of physical training and blueberry extract, either alone or in combination, on the progression of pulmonary hypertension and its impact on LV function.

## 5. Conclusions

Our findings indicate that blueberry extract and moderate-intensity resistance training, when administered during the progression of MCT-induced severe PAH in rats, prevent oxidative imbalance and structural deterioration in the LV. These protective effects contribute to the maintenance of LV function, highlighting the potential of these interventions in mitigating cardiac impairments associated with PAH.

## Figures and Tables

**Table 1:** Blueberry extract composition.

Compound	Concentration
Total phenolic compounds (mg GAE/g)	38.89
Total flavonoid compounds	
Cyanidin (µg/mL)	219.21 ± 5.38
Malvidin (µg/mL)	128.68 ± 1.27
Rutin	ND
Gallic acid	ND
Catechin	ND
Epicatechin	ND

GAE—gallic acid equivalent; ND—not detected.

**Table 2:** Percentage (%) of inflammation in the left ventricle of healthy Wistar rats, hypertensive rats, and hypertensive rats treated with blueberry extract, resistance training, or their combination.

Grade	SC	SH	SHB	EH	EHB
0	2 (28.57%)	0 (0.00%)	0 (0.00%)	0 (0.00%)	0 (0.00%)
I	4 (57.14%)	0 (0.00%)	0 (0.00%)	0 (0.00%)	0 (0.00%)
II	0 (0.00%)	0 (0.00%)	0 (0.00%)	0 (0.00%)	2 (28.57%)
III	1 (14.20%)	0 (0.00%)	2 (28.57%)	1 (14.20%)	1 (14.20%)
IV	0 (0.00%)	0 (0.00%)	5 (71.42%)	6 (85.71%)	4 (57.14%)
V	0 (0.00%)	7 (100.00%)	0 (0.00%)	0 (0.00%)	0 (0.00%)

Inflammation was assessed using a semiquantitative scale, where 0 represents no lesions, I represents less than three foci, II represents more than three foci, III indicates mild inflammation, IV indicates moderate inflammation, and V indicates severe inflammation. The experimental groups are as follows: SC (sedentary control), SH (sedentary hypertensive), SHB (sedentary hypertensive treated with blueberry extract), EH (hypertensive treated with resistance training), and EHB (hypertensive treated with blueberry extract and resistance training). *n* = 7 animals/group.

## Data Availability

The data will be shared upon reasonable request to the corresponding author due to ethical restrictions related to the use of animals in research.

## Supplementary Materials

The following supporting information can be downloaded at: https://www.mdpi.com/article/10.3390/nu17071145/s1, Figure S1: Effects of blueberry extract and resistance training on pulmonary artery resistance and on the left ventricle remodeling. (A) Representative images of pulmonary artery flow. (B) Representative photomicrographs of left ventricle tissue stained with hematoxylin and eosin (scale bar: 200 μm). Black arrows: inflammatory infiltrate; red arrows: blood vessel.
