# Supplementary material for: Blueberry Extract and Resistance Training Prevent Left Ventricular Redox Dysregulation and Pathological Remodeling in Experimental Severe Pulmonary Arterial Hypertension
Source: Nutrients. 2025 Mar 26;17(7):1145. doi: 10.3390/nu17071145 (PMC11990098; doi:10.3390/nu17071145)
Supplement: Supplementary file 1 [file nutrients-17-01145-s001.zip › nutrients-3530858-supplementary.pdf]

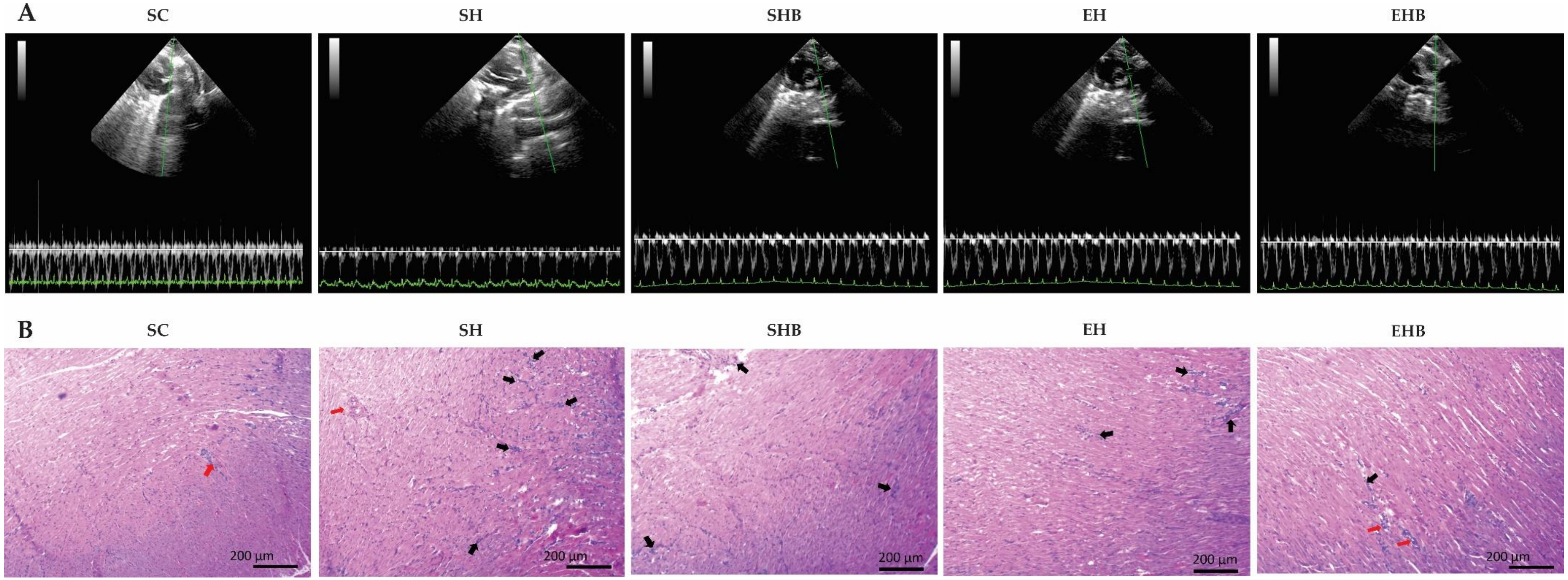

Supplementary Figure S1. Effects of blueberry extract and resistance training on pulmonary artery resistance and on the left ventricle remodeling. (A) Representative images of pulmonary artery flow. (B) Representative photomicrographs of left ventricle tissue stained with hematoxylin and eosin (scale bar: 200 µm). Black arrows: Inflammatory infiltrate; red arrows: blood vessel.
